# Searching for bridges between psychopathology and real-world functioning in first-episode psychosis: A network analysis from the OPTiMiSE trial

**DOI:** 10.1192/j.eurpsy.2022.25

**Published:** 2022-06-10

**Authors:** Francesco Dal Santo, Eduardo Fonseca-Pedrero, María Paz García-Portilla, Leticia González-Blanco, Pilar A. Sáiz, Silvana Galderisi, Giulia Maria Giordano, Julio Bobes

**Affiliations:** 1 Área de Psiquiatría, Universidad de Oviedo, Oviedo, Spain; 2 Servicio de Salud del Principado de Asturias (SESPA), Oviedo, Spain; 3 Instituto de Investigación Sanitaria del Principado de Asturias (ISPA), Oviedo, Spain; 4 Instituto de Neurociencias del Principado de Asturias (INEUROPA), Oviedo, Spain; 5 Centro de Investigación Biomédica en Red de Salud Mental (CIBERSAM), Madrid, Spain; 6 Department of Educational Sciences, University of La Rioja, Logroño, Spain; 7 Department of Psychiatry, University of Campania “Luigi Vanvitelli”, Naples, Italy

**Keywords:** first-episode psychosis, network analysis, psychopathology, real-world functioning, schizophrenia

## Abstract

**Background:**

Network analysis has been used to explore the interplay between psychopathology and functioning in psychosis, but no study has used dedicated statistical techniques to focus on the bridge symptoms connecting these domains. The current study aims to estimate the network of depressive, negative, and positive symptoms, general psychopathology, and real-world functioning in people with first-episode schizophrenia or schizophreniform disorder, focusing on bridge nodes.

**Methods:**

Baseline data from the OPTiMiSE trial were analyzed. The sample included 446 participants (age 40.0 ± 10.9 years, 70% males). The network was estimated with a Gaussian graphical model, using scores on individual items of the positive and negative syndrome scale (PANSS), the Calgary depression scale for schizophrenia, and the personal and social performance scale. Stability, strength centrality, expected influence (EI), predictability, and bridge centrality statistics were computed. The top 20% scoring nodes on bridge strength were selected as bridge nodes.

**Results:**

Nodes from different rating scales assessing similar psychopathological and functioning constructs tended to cluster together in the estimated network. The most central nodes (EI) were Delusions, Emotional Withdrawal, Depression, and Depressed Mood. Bridge nodes included Depression, Conceptual Disorganization, Active Social Avoidance, Delusions, Stereotyped Thinking, Poor Impulse Control, Guilty Feelings, Unusual Thought Content, and Hostility. Most of the bridge nodes belonged to the general psychopathology subscale of the PANSS. Depression (G6) was the bridge node with the highest value.

**Conclusions:**

The current study provides novel insights for understanding the complex phenotype of psychotic disorders and the mechanisms underlying the development and maintenance of comorbidity and functional impairment after psychosis onset.

## Introduction

Psychotic disorders are severe, complex, multifactorial mental disorders characterized by heterogeneous psychopathological features. These include positive, negative, cognitive, and affective symptoms, and disorganized behaviors [1, 2]. None of these is pathognomonic of the illness, and individuals can present varying degrees of severity in the different symptomatologic areas [3]. Several studies, for example, have highlighted the importance of mood symptoms, given their high prevalence in both prodromal and clinical psychosis [4]. In fact, it has been estimated that up to 80% of individuals experience a clinically significant depressive episode during the early phase of the mental disorder [4]. While previous models have conceptualized depressive and psychotic symptoms as distinct clinical features of the mental disorder, existing research has shown difficulties differentiating between these symptomatologic domains [5], highlighting their close relationship, as in the case of negative symptoms and depression [3].

Moreover, despite significant advances in pharmacological and psychological treatments, psychotic disorders still rank among the leading causes of disability worldwide, with undeniably substantial burdens for the affected individuals and their families and caregivers [6], as well as for the health and welfare systems, especially in younger age groups [7]. However, functional impairment still represents a challenge for clinicians and researchers, this having also been suggested as a resistance criterion by some authors [8]. A recent meta-analysis found a functional recovery rate of 38% in first-episode psychosis (FEP) and observed a tendency toward stabilization after the first 2 years of illness [9]. Different areas of functioning can be affected. For example, social functioning is often impaired, as people with this disorder tend to interact less with others or do so in a socially inappropriate way, which may reduce the willingness of others to engage with them [1]. Thus, identifying the predictors of poor functioning should be a priority and could inform the development of new targets to better assess and manage individuals with FEP. However, existing research has shown heterogeneous results in this population, suggesting that functional outcomes could be related to diverse psychopathological characteristics [10].

In this context, emerging network analysis techniques, which aim to suggest new ways of modeling and understanding psychopathological processes [11–13], could help disentangle the complex and dynamic interplay between symptoms and functional outcomes. This approach focuses on conceptualizing symptoms as mutually interacting and often reciprocally reinforcing elements of a complex network rather than interpreting them as a function of a latent disorder [11, 14, 15]. So, from this perspective, mental disorders are presumed to arise from direct interactions between symptoms in a network architecture [15].

One of the main reasons for the increasing popularity of these theories is the inadequacy of the traditional categorical approach, which has led to a simplified and incomplete vision of mental problems [11]. Moreover, network techniques offer multiple methodological advantages over other methods, including the possible verification of simultaneous relations among variables [16] and the lack of need for a priori assumptions regarding relationships among the variables or the selection of predictors, mediators, and outcome measures [17]. Furthermore, this paradigm shift may also have important clinical implications and could promote a personalized and integrated approach to the treatment of psychosis [17]. For example, it could reveal novel therapeutic targets (e.g., influential nodes susceptible to deactivation) that might otherwise go unnoticed with traditional methodology and thus be neglected by clinicians in a real-life setting.

It therefore comes as no surprise that, in recent years, network analysis has been applied to the study of correlates of functioning in both established schizophrenia [17, 18] and FEP [16, 19, 20]. Using this approach, Galderisi and colleagues highlighted the critical role of real-world functioning, primarily the everyday life skills domain, in a sample of community-dwelling individuals with schizophrenia, where functioning nodes were linked to different clinical correlates such as disorganization, expressive deficits, and avolition [17]. While the overall network structure of the sample was similar at the 4-year follow-up, a further analysis revealed a very sparse network, with real-life functioning disconnected from other nodes in the recovered subgroup [18], emphasizing the dynamic nature of these interactions and their relevance to clinical and functional recovery. Regarding FEP, Chang et al. [19] found that psychosocial functioning was strongly associated with amotivation, moderately with positive symptoms, and only weakly with other psychopathological variables. However, the study sample was constituted of participants aged 26–55 years, making these findings less generalizable to first-episode cohorts with younger age of illness onset. On the other hand, another study recently observed several connections between functioning problems and psychopathology, including hallucinations, conceptual disorganization, and depression [20].

Nonetheless, generalizability of previous research is problematic, mainly due to methodological differences. Some studies, for example, computed the network by introducing the total of all scores on rating scales (or all subscale scores) [17–19], limiting an examination of the role played by individual symptoms in determining functional impairment. Conversely, despite their use of individual items, other authors did not introduce the full list of PANSS items or a specific instrument for depression [16]. Also, these studies included participants from single nations. This could be a limitation because, for example, different cultural contexts may vary in the degree to which they accept particular symptoms (e.g., hallucinations and magical thinking) as normative experiences [21].

Moreover, previous network analysis studies focusing on functional outcomes have not made use of dedicated statistical procedures to identify so-called “bridge nodes” [22]. These nodes represent the key connection points between different groups of nodes in a network (e.g., groups of nodes that belong to specific psychopathological or functioning domains) and deserve special attention for their possible contribution to the onset and maintenance of comorbid conditions in mental disorders [12]. From a translational perspective, bridge nodes might be used to develop targeted interventions, and deactivating symptoms based on their bridge strength rather than on other centrality measures may constitute an effective strategy to prevent comorbidity [22].

Thus, the purposes of the current study are (a) to use a network approach to shed light on the interplay among depressive, negative, and positive symptoms, general psychopathology, and deficits in personal, social, and occupational functioning in people with first-episode schizophrenia or schizophreniform disorder and (b) to statistically identify the bridge nodes of the estimated network.

## Method

### Study design and participants

This study is a secondary analysis of data from the Optimization of Treatment and Management of Schizophrenia in Europe (OPTiMiSE) trial, for which a detailed description of the rationale and methodology can be found elsewhere [23].

Individuals with FEP were recruited at the participating centers, which included 27 general hospitals and psychiatric specialty clinics in 14 European countries (Austria, Belgium, Bulgaria, Czech Republic, Denmark, France, Germany, Italy, the Netherlands, Poland, Romania, Spain, Switzerland, and the UK) and Israel. Eligible participants aged 18–40 years who met the Diagnostic and Statistical Manual of Mental Disorders (4th edition) criteria for schizophrenia, schizophreniform disorder, or schizoaffective disorder were recruited. Diagnoses were confirmed by the Mini-International Neuropsychiatric Interview-Plus [24].

They were excluded if: (a) more than 2 years had elapsed between the onset of psychosis and enrollment; (b) they had been treated with any antipsychotic medication for more than 2 weeks in the previous year or a total of 6 weeks or more lifetime; (c) they had a previous history of intolerance to one of the study drugs; (d) they met any of the contraindications for any of the study drugs; (e) they were coercively treated or represented by a legal guardian, or both, or in legal custody; or (f) they were pregnant or breastfeeding at the study time.

This study was conducted in accordance with the ethical principles of the Declaration of Helsinki, and each country obtained ethics approval. All participants received information about the purposes and protocol of the study and signed the informed consent before any procedures were performed.

For the current analysis, we employed data collected as part of phase 1 of the original study (open label amisulpride treatment at a daily dose of 200–800 mg), in which a total of 446 participants were enrolled from an initial sample of 481 subjects who were assessed for eligibility and signed an informed consent [25].

### Measures

After completing an initial screening visit to assess eligibility, baseline data were collected, including sociodemographic variables, diagnoses, current treatments, and rating scales.

The psychopathological assessment included the positive and negative syndrome scale (PANSS) [26] to characterize psychotic symptoms and the Calgary Depression Scale for Schizophrenia (CDSS) [27] to assess depression. The clinical global impression-schizophrenia [28] was used to evaluate severity of illness.

Finally, real-world functioning was assessed with the personal and social performance (PSP) scale [29]. The PSP scale is a clinician-rated instrument that evaluates four areas of functioning: (a) Socially Useful activities, (b) Personal and Social Relationships, (c) Self-care, and d) Disturbing and Aggressive behavior. These subscores range from 0 to 6, where higher scores indicate worse functioning. A total score (ranging from 0 to 100) is also calculated, with higher scores corresponding to better personal and social functioning.

### Data analyses

First, the sociodemographic characteristics of the sample and the descriptive statistics of all measures used were analyzed, expressing the results with means, standard deviations (SD), and percentages.

Second, the network of psychosis phenotype, depression symptoms, and functional outcomes was estimated. A Gaussian graphical model [30] was used for this purpose. In this network, the scores on the individual items of the instruments were used instead of the scale or subscale total scores. Therefore, the analysis was performed with a total of 43 nodes: the 30 items of the PANSS, the nine items of the CDSS, and the four items of the PSP.

The details of network analysis have been documented in previous publications [31, 32]. A network consists of nodes (study variables, such as item scores on each measurement instrument) and edges (estimated statistical relationships among variables). We used partial correlations: if two nodes are connected in the resulting graph via an edge, they are statistically related after controlling for all other variables in the network; if they are unconnected, they are conditionally independent. The least absolute shrinkage and selection operator (LASSO) procedure was used to limit the number of spurious connections among nodes [30]. This regularization method applies a penalty to small edges, shrinking them to zero and thus dropping them out of the model, and returning a network model that is more stable and easier to interpret. For the layout, we used the Fruchterman–Reingold algorithm, placing the strongly connected nodes closer to each other and the less connected nodes far apart [32].

In keeping with previous studies examining networks [33], we estimated two inference measures: strength centrality and expected influence (EI). Strength centrality is the sum of the correlations of one node to all other nodes of the network. High values reflect great centrality of the node in the network. EI identifies the most important nodes within a network graph [34]. We used EI along with strength centrality in order to avoid possible problems associated with centrality measures [35, 36]. It is noteworthy that strength centrality uses the sum of absolute weights (i.e., negative edges are turned into positive edges before summing), so the interpretation could be distorted if negative edges are present (as in the present article). On the other hand, EI takes into account negative associations among nodes and can assume negative values. If the EI value of a node is negative, changes in the node should produce network changes in the opposite direction (e.g., decreases in node activation should lead to increases in overall network activation) [34].

Network stability and accuracy were estimated using the bootstrapping analysis in the R *bootnet* package [31].

Finally, further analyses were performed to identify the bridge nodes. Two bridge centrality statistics (bridge strength and bridge betweenness) were estimated with the bridge function in the *networktools* package [37]. We selected the top 20% scoring nodes on bridge strength as bridge nodes, following the methods from previous research [22].

We used IBM SPSS Statistics for Windows, Version 22.0 [38], JASP (https://jasp-stats.org/), and *R* [39] to perform data analyses.

## Results

### Participants and descriptive statistics

A total of 446 participants enrolled and started phase 1. Sociodemographic, clinical, and psychometric characteristics of the sample as well as the descriptive statistics of all measures are depicted in Table 1.

**Table 1.** Sociodemographic data and clinical and functional assessment of the sample (*n* = 446).

	Whole sample (*n* = 446)
Age, years (mean; SD)	25.96 (5.99)
Sex, male	312 (70%)
Race	
White	386 (86.5%)
Others	60 (13.5%)
Education, years (mean; SD)	12.27 (2.95)
Employment status	
Employed or student	185 (41.5%)
Unemployed	261 (58.5%)
Disease type	
Schizophreniform disorder	190 (42.6%)
Schizoaffective disorder	27 (6.1%)
Schizophrenia	229 (51.3%)
Clinical scores (mean; SD)	
PANSS positive	20.16 (5.53)
PANSS negative	19.39 (7.11)
PANSS GP	38.61 (9.84)
PANSS total score	78.15 (18.70)
CGI-SCH severity	5.50 (0.93)
CDSS total	4.53 (4.58)
PSP—activities	3.94 (1.07)
PSP—relationships	3.54 (1.08)
PSP—self-care	2.20 (1.18)
PSP—aggressive behaviors	1.81 (1.13)
PSP—total score	48.37 (15.33)

*Note:* Data are expressed as mean (SD) or *n* (%).

*Abbreviations:* CDSS, Calgary Depression Scale for Schizophrenia; CGI-SCH, clinical global impression-schizophrenia; GP, general psychopathology; PSP: Personal and Social Performance Scale; PANSS, Positive and Negative Syndrome Scale.

### Network structure of psychosis phenotype

**Figure 1.**
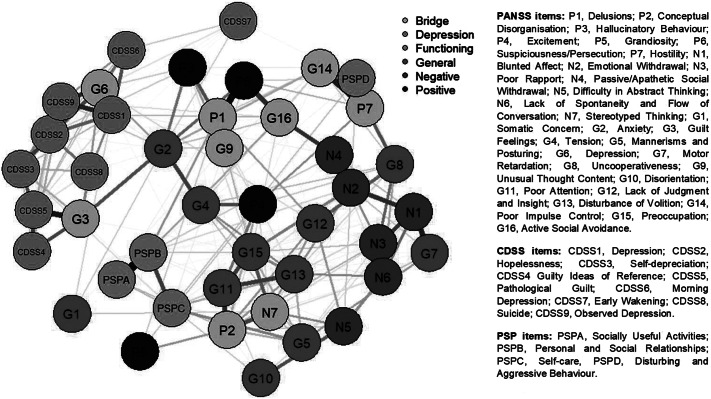
Estimated network for psychosis phenotype, depression symptoms, and real-life functioning. CDSS: Calgary Depression Scale for schizophrenia; G: PANSS, general psychopathology dimension; N: PANSS, negative psychosis dimension; PSP: personal and social performance; P: PANSS, positive psychosis dimension. Numbers represent item numbers in the scale; blue edges represent positive associations; red edges represent negative associations. Thickness and saturation of edges indicate the strength of these associations.

The estimated psychosis network showed a high degree of interconnectedness between nodes (see Figure 1). Most of the associations between edges were positive (see Figure 1).

**Figure 2.**
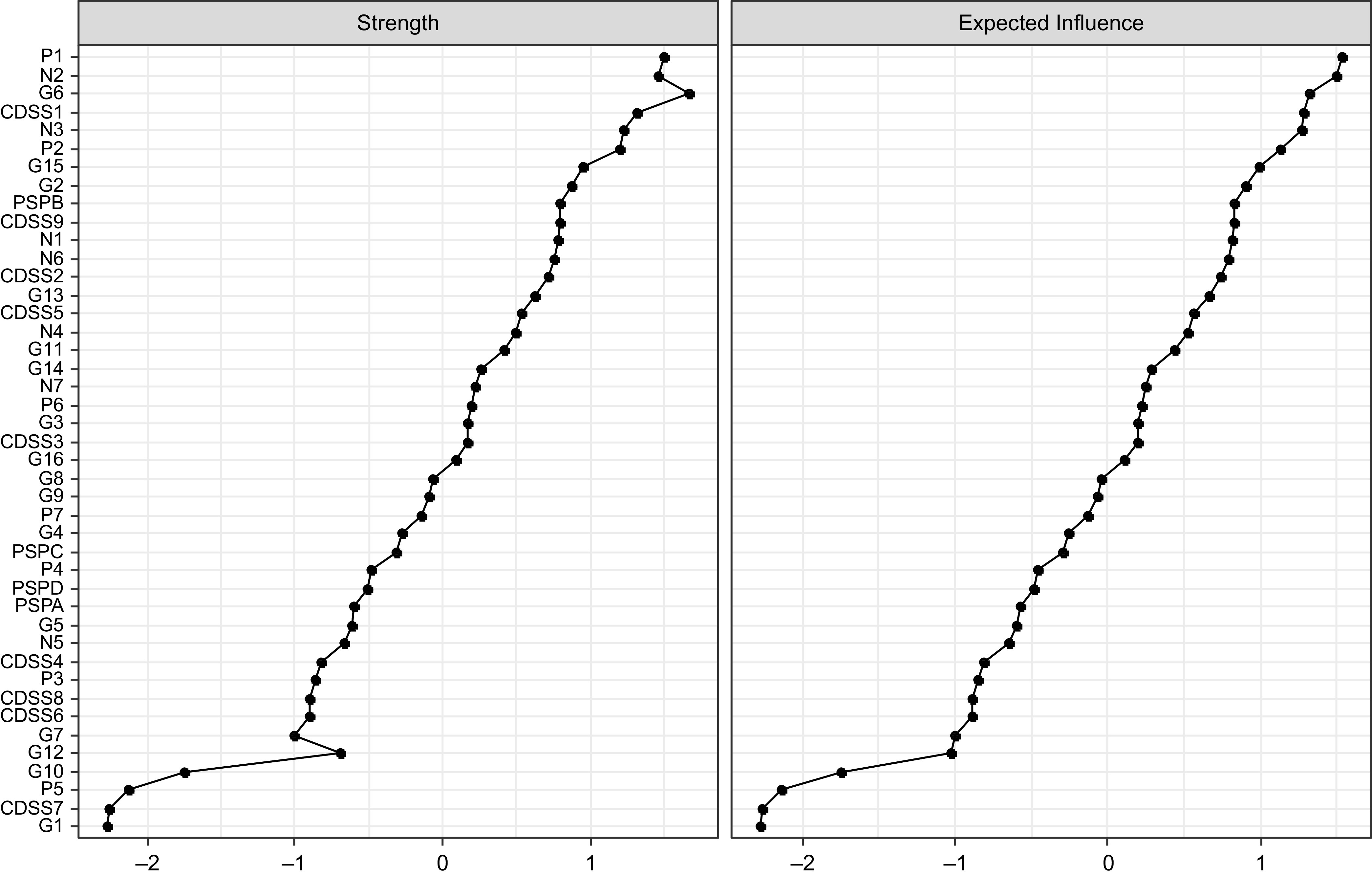
Inference measures of the estimated psychosis network. CDSS: Calgary Depression Scale for Schizophrenia; G: PANSS, general psychopathology dimension; N: PANSS, negative psychosis dimension; PSP: personal and social performance; P: PANSS, positive psychosis dimension. Numbers represent item numbers in the scale.

Strength centrality and standardized EI values are depicted in Figure 2. The most central nodes in terms of strength were Depression (G6), Delusions (P1), Emotional Withdrawal (N2), and Depressed Mood (CDSS1). The most central nodes in terms of EI were Delusions (P1), Emotional Withdrawal (N2), Depression (G6), Depressed Mood (CDSS1), and Poor Rapport (N3). Somatic Concern (G1), Early Wakening (CDSS7), Grandiosity (P5), and Disorientation (G10) showed a negative value in this centrality index.

The adaptive LASSO network showed that items from different rating scales but assessing similar psychopathological and functioning constructs tended to cluster together (see Figure 1).

Depression symptoms formed a cluster of nodes with a high degree of interconnectedness, with the exception of the Early Wakening node (CDSS7), which showed a direct connection with only the Morning Depression item (CDSS6) of the CDSS. In addition, the CDSS nodes were positively and closely related to the Guilty Feelings (G3) and Depression (G6) nodes of the PANSS general psychopathology subscale and not clearly related to psychosis symptom nodes. Furthermore, these nodes were separate from psychosocial functioning as measured with the PSP.

Regarding psychosis phenotype symptoms as measured with the PANSS, the items of the negative dimension tended to be consistently more strongly related compared with the items that make up the positive dimension, revealing a cluster made up of the Blunted Affect (N1), Emotional Withdrawal (N2), Poor Rapport (N3), Passive/Apathetic Withdrawal (N4), Lack of Spontaneity, and Flow of Conversation (N6) nodes. The psychotic positive cluster included Delusions (P1), Hallucinatory Behavior (P3), and Suspiciousness/Persecution (P6). Mixed results were found for the items of the PANSS general psychopathology domain, scattered throughout the network and associated with different psychopathological domains. This is exemplified by the Unusual Thought Content (G9) and Motor retardation (G7) nodes, which were closely related to the positive and negative groups of nodes, respectively. On the other hand, disorganized symptoms congregated to form a cluster of items from the three PANSS subscales, which included the Conceptual Disorganization (P2), Difficulty in Abstract Thinking (N5), Stereotyped Thinking (N7), Preoccupation (G15), Disturbance of Volition (G13), Poor Attention (G11), Disorientation (G10), and Mannerisms and Posturing (G5) nodes. Lack of Insight (G12) fluctuated between positive and disorganized domains.

With respect to real-world functioning nodes, the network depicted a clear separation between two dimensions. The first one was related to psychosocial functioning and incorporated Socially Useful Activities (PSP-A), Personal and Social Relationships (PSP-B), and Self-care (PSP-C). This group of nodes maintained connections with both the positive and disorganized clusters of the network. By contrast, the Disturbing and Aggressive Behavior item of the PSP (PSP-D) was strongly associated with the Hostility (P7) and Poor Impulse Control (G14) nodes.

### Bridge nodes and bridge centrality measures

**Figure 3.**
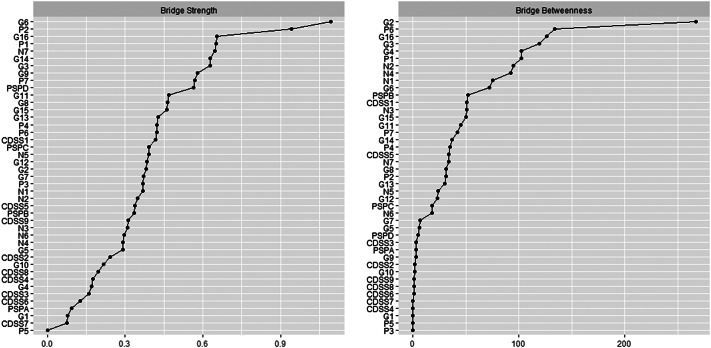
Bridge centrality measures of the estimated psychosis network. CDSS: Calgary Depression Scale for Schizophrenia; G: PANSS, general psychopathology dimension; N: PANSS, negative psychosis dimension; PSP: personal and social performance; P: PANSS, positive psychosis dimension. Numbers represent item numbers in the scale.

Bridge centrality statistics (bridge strength and bridge betweenness) are reported in Figure 3.

The top 20% scoring nodes on bridge strength were Depression (G6), Conceptual Disorganization (P2), Active Social Avoidance (G16), Delusions (P1), Stereotyped Thinking (N7), Poor Impulse Control (G14), Guilty Feelings (G3), Unusual Thought Content (G9), and Hostility (P7). Most of the bridge nodes belonged to the general psychopathology subscale of the PANSS (see Figure 1).

Depression (G6) and Guilty Feelings (G3) represented the bridge between depressive symptoms and the rest of the network, especially through the shared connection with the Anxiety node (G2), which showed the highest value of bridge betweenness. Hostility (P7) and Poor Impulse Control (G14) nodes constituted the bridge between the positive symptoms cluster and the Disturbing and Aggressive Behavior item of the PSP (PSP-D). Delusions (P1), the most central node in terms of EI, emerged as a bridge between the positive dimension and psychosocial functioning. Active Social Avoidance (G16) connected the positive and negative dimensions and also showed a weaker link with Personal and Social Relationships (PSP-B), while Unusual Thought Content (G9) linked the positive dimension with the disorganized symptoms. The latter group also formed bridges with psychosocial functioning and the negative dimensions through Conceptual Disorganization (P2) and Stereotyped Thinking (N7).

### Network stability and accuracy analysis

The results of the stability and accuracy analysis [31] indicated that the psychosis network was accurately estimated, with adequate confidence intervals around the edge weights. Details are accessible in Supplementary Figures S1 and S2.

## Discussion

The present study was designed to explore the interplay between psychosis phenotype, general psychopathology, depression, and real-world functioning in people with FEP and represents one of the first attempts to assess these interactions at symptom level in this clinical population. Novel network analysis techniques, such as identification of bridge nodes, were employed to identify central nodes. The analysis yielded a stable and accurately estimated network.

### Structure of the network

The topological structure of this network offers several interesting findings and highlights the complex nature of psychopathology in FEP. One of the main findings of our analysis is that the items belonging to different rating scales but assessing similar psychopathological constructs tend to cluster together in a manner not always consistent with the original structure of the scales. This is especially evident in the case of the PANSS: while the nodes from the CDSS tended to form a well-connected group, the items from the Positive, Negative, and General Psychopathology subscales sparsely mixed with each other or with the other items of the network. Taken together, these results seem to indicate that this three-dimensional solution may prove to be unsatisfactory when describing psychopathology in people with FEP. In this sense, it is interesting to observe a partial overlap of the clusters of nodes identified in our network with earlier five-factor solutions [40]. However, those approaches produced very few models with acceptable fits, making it advisable to explore new methods [41]. In this regard, the emergence and refining of network analysis techniques could offer an additional tool to capture the dimensional nature of psychotic symptoms, focusing not only on identification of symptom clusters but above all on their connecting (and, by extension, activating) patterns.

It is somewhat surprising that our findings are contrary to other network analyses, which have observed that nodes that belong to the same subscale are highly interconnected [5, 42, 43]. However, it should be noted that a direct comparison of our findings with those of previous studies is not feasible in most of the cases due to methodological differences. First, some of the existing studies included the total score (or subscale total scores) of the rating scales instead of computing the network via individual symptoms [17–19, 44]. Despite considering individual symptoms as network nodes, other authors did not include general psychopathology in their analyses [5, 43] or selected a reduced number of items from the PANSS general psychopathology subscale [16, 42]. Interestingly, a recent network analysis of a sample of individuals at clinical high risk for psychosis or with recent-onset psychosis highlighted the importance of general psychopathology as a potential trigger of the pathway from negative life events to the expression of psychotic symptomatology [45], while other authors have found general psychopathology subscale score to be the node with the highest strength [44]. Therefore, based on our findings, we recommend including the PANSS general psychopathology subscale in network models, along with the positive and negative subscales. We believe that this approach could prove useful to better capture the diverse psychopathological presentation of FEP beyond the positive/negative symptom duality.

Regarding functioning, the high degree of interconnectedness between Socially Useful Activities, Personal and Social Relationships, and Self-care found in our network analysis is consistent with earlier findings [17, 18]. However, in contrast, the functioning nodes were not the most important in our network in terms of strength centrality and standardized EI values, supporting evidence from previous observations in FEP samples [16, 19]. A possible explanation for these results may lie in the differences in the samples studied. First, the former studies by Galderisi and colleagues were not conducted in FEP, suggesting that the network structure could change over the course of the illness. Moreover, they focused on stabilized community-dwelling individuals who could still exhibit significant functional impairment despite resolution of the acute symptom exacerbation, resulting in the lower relative importance of the psychotic symptoms in their network. On the other hand, the Disturbing and Aggressive Behaviors node was associated with other symptoms, namely Hostility (P7) and Poor Impulse Control (G14), and seems to be better conceptualized as a separate and specific construct.

### Bridges nodes

Another initial objective of the project was to identify bridge nodes using a specific statistical analysis. If we consider the network a dynamic representation of psychopathology [13] and its impact on functioning, specific interventions targeting these bridge nodes could lower the degree of activation of other nodes and therefore lead to better overall outcomes. Given that the majority of the studies relied on visual inspection rather than more refined statistical techniques to identify these key symptoms, the current study provides novel insights for understanding the complex phenotype of psychotic disorders and the mechanisms underlying the development and maintenance of comorbidity and functional impairment after psychosis onset.

When examining the bridge strength measure, we found that Depression (G6) was the bridge node with the highest value. This node, which was also one of the most important in terms of EI, connected the symptoms of the CDSS to the rest of the network along with Guilty Feelings (G3), and their bridge function acted through an interesting shared pathway via the Anxiety node (G2). The significance of these nodes in our network is not surprising, as several reports have stressed the high rates of depression in schizophrenia, especially during the early phase of the disorder [4, 46], with potential serious outcomes such as suicidal behaviors [47]. In keeping with the present results, a recent longitudinal study in an FEP sample found that depression was one of the most important nodes in their network [43] and further revealed that it maintained its central role at the 12-month follow-up despite the amelioration of psychotic symptoms [46]. The topographic proximity of depressive to positive symptoms in our network is also consistent with previous findings [5, 43, 48] and constitutes an alert that potential worsening of the affective domain could translate to a flare up of psychotic symptoms [43] and vice versa. However, their networks did not include general psychopathology, so the present study raises the possibility that other nodes may underlie these relationships. Anxiety (G2), which showed the highest value of bridge betweenness in our network and is considered among the least studied features of schizophrenia [49], offers a clear example of a potential treatment target to avoid the spread of contagion between affective symptoms and the psychotic dimension.

On the other side of the network, Delusions (P1), unsurprisingly the most important node of our estimated network, showed a double connection with Personal and Social Relationships (PSP-B) as an indirect pathway through Active Social Avoidance (G16) emerged, in addition to the direct link between P1 and PSP-B. Moreover, Active Social Avoidance (G16) mediated the pathway from positive to negative symptom clusters. This combination of findings may have different clinical implications. First, due to its influence on the network and its connecting pattern, a worsening of delusions may potentially lead to an overall activation of the network, directly reinforcing other psychopathological nodes as well deteriorating functional abilities. Eradicating positive symptomatology, for example, by following evidence-based treatment schemes [25], seems therefore crucial to prevent inter-symptom contagion. Second, the impact of positive symptoms on functioning appears to be partly related to a specific subtype of social avoidance, which also bridges the path between positive and negative symptoms. A double reading can be derived from these findings: while early intervention on positive symptoms may prevent social isolation and, by extension, avoid the emergence of negative symptoms, if the aforementioned pathways are followed in reverse, promoting better social interactions, this could facilitate clinical recovery in FEP, as suggested by previous research [50]. With this in mind, clinicians should carefully distinguish active social avoidance from passive/apathetic social withdrawal, as it may require other kinds of interventions [51, 52], for example, targeting theory of mind processes with metacognitively oriented psychotherapies [53].

With respect to the bridge nodes linking psychosocial functioning with the disorganized dimension, our findings support the existing literature on applying the network approach, which stresses the impact of disorganized symptoms on functional outcomes in individuals with established schizophrenia [17] and highlights the prominent role of conceptual disorganization as a connector between functioning and clinical symptoms in FEP [16]. Interestingly, another study previously found that conceptual disorganization could have an impact on community activities up to twice as high as core symptoms such as delusions and avolition [54]. Moreover, both Conceptual Disorganization (P2) and Stereotyped Thinking (N7) were recently signaled as key bridge nodes in a network from a sample of people with FEP [43], although that study did not include a functional assessment. Hence, while formal thought disorder risk may be under-recognized during the first stages of illness, its early detection could offer a novel treatment target [43] with potential beneficial effects on both clinical and functional recovery.

In the case of the Disturbing and Aggressive Behaviors node (PSP-D), our network model revealed several direct and indirect connections with a broad range of psychosis symptoms on the PANSS subscales. This reflects the complex interplay between psychopathology and aggressive behavior in early-stage psychosis and corroborates the findings of existing research studies, which found relationships with delusions [55], uncooperativeness [56], impulsivity [57], excitement [58], lack of insight [58], and negative symptoms [59], among others. According to the present results, a comprehensive assessment and management of these symptoms could facilitate prevention strategies and directly or indirectly reduce the risk of aggression due to diminished Poor Impulse Control (G14) and Hostility (P7). These findings are important not only in light of the common occurrence of disturbing and aggressive behaviors in people with FEP but also because of the drop-off in aggression rates observed in individuals with regular follow-up by mental health services after a first psychotic episode [59, 60]. However, it should be mentioned that the study sample was characterized by low rates of disturbing and aggressive behavior, as discussed elsewhere [60].

Finally, it is worth mentioning that, to the best of our knowledge, our study provides the first cross-national network model of the inter-relationships among psychopathology, depression, and functioning in FEP. Although this collaborative effort could translate into better overall generalizability of the current findings, it should also be noted that specific social, cultural, and historical aspects may play a modulating role and result in symptom networks differing partially across place and time [15]. For instance, differences between countries have been observed in previous multinational network analyses of schizotypal personality traits [33]. Different cultural contexts may also vary in identifying particular symptoms as anomalous or unusual [21], possibly determining, for example, a different impact on the level of social functioning of young people with FEP. However, the characteristics and the size of the sample limited our possibility of performing in-depth country-based analyses. Nevertheless, since the vast majority of the participating centers are located in European countries that share a similar cultural and social background, we would not expect substantial differences in this regard. More research is therefore warranted to address this issue, especially longitudinal cross-cultural studies that could provide further knowledge in the field of psychosis about the impact of cultural and social environment on the dynamic evolution of these interactions over time.

### Strengths and limitations

It is worth pointing out some strengths of our study, such as the size of the collected sample, the multicenter nature of the study, and the wide-ranging psychopathological and functional variables assessed. Moreover, the strict inclusion criteria of the trial allow the control of a series of confounders. Furthermore, the network model was designed to consider individual symptoms as nodes, the stability analyses indicate that this network is accurately estimated, and novel inference measures such as EI and predictability were computed. Finally, bridge centrality statistics were calculated to identify bridge nodes, instead of relying on visual inspection as previous studies did. Also, while previous research has mainly employed bridge analysis to explore comorbidity between different mental disorders, the current study focused on various aspects of the phenotype of psychotic disorders.

However, the reader should be aware of some limitations when interpreting the current findings. Selection bias is perhaps the main potential concern, as participants were recruited to take part in a randomized controlled trial. For example, the exclusion of individuals coercively treated or represented by a legal guardian, who could represent a group with worse overall symptom severity and functioning, could limit the generalizability of the current findings. Also, the cross-sectional analysis performed suffers from well-known drawbacks. Furthermore, the clinical evaluation lacked an intelligence quotient evaluation, as well as a dedicated cognitive assessment, although the direct impact of cognition on real-world functioning is at present still a matter of debate.

## Conclusions

The findings of this investigation indicate that it does not seem appropriate to simplify and compartmentalize the psychopathological presentation of FEP using the original structure of the three PANSS subscales and that innovative techniques such as network analysis, focusing on the interactions between individual symptoms, could prove useful to better capture the complex interplay between symptoms and functional outcomes.

The current research also statistically identified several bridge nodes, whose deactivation could inhibit the cascade of self-reinforcing interactions between symptoms and functioning nodes in the estimated network. Although interventions on positive symptoms are crucial in FEP, we stress the importance of other symptoms such as anxiety, depression, disorganization, impulsivity, active social avoidance, and social relationships. Too often neglected by clinicians, these bridge nodes need to be carefully assessed during the early stages of the illness and should constitute preferential intensive treatment targets to reduce comorbidity and preserve real-world functioning. In accordance with the network theory [15], tailored interventions targeting these nodes should involve multidisciplinary approaches, including pharmacological and psychosocial strategies.

## Data Availability

The data that support the findings of this study are available from the corresponding author, M.P.G-P., upon reasonable request. Restrictions in relation to potentially person identifiable information apply.
